# Cancer testis antigen Sperm Protein 17 as a new target for triple negative breast cancer immunotherapy

**DOI:** 10.18632/oncotarget.20102

**Published:** 2017-08-10

**Authors:** Leonardo Mirandola, Elisa Pedretti, Jose A. Figueroa, Raffaella Chiaramonte, Michela Colombo, Caroline Chapman, Fabio Grizzi, Federica Patrinicola, W. Martin Kast, Diane D. Nguyen, Rakhshanda Layeequr Rahman, Naval Daver, Peter Ruvolo, Sean M. Post, Robert S. Bresalier, Maurizio Chiriva-Internati

**Affiliations:** ^1^ Kiromic Inc., Houston, TX, USA; ^2^ Department of Health Sciences, Universita’ degli Studi di Milano, Milano, Italy; ^3^ Bowel Cancer Screening Programme, Eastern Hub Queens Medical Centre, Nottingham University Hospitals, Nottingham, UK; ^4^ Department of Immunology & Inflammation, Humanitas Clinical & Research Center, Milan, Italy; ^5^ Departments of Obstetrics & Gynecology and Molecular Microbiology & Immunology, University of Southern California, Los Angeles, CA, USA; ^6^ Texas Tech University Health Sciences Center, Amarillo Breast Center of Excellence, Amarillo, TX, USA; ^7^ Department of Leukemia, The University of Texas MD Anderson Cancer Center, Houston, TX, USA; ^8^ Department of Gastroenterology, Hepatology and Nutrition, Division of Internal Medicine, The University of Texas MD Anderson Cancer Center, Houston, TX, USA; ^9^ Department of Lymphoma & Myeloma, The University of Texas MD Anderson Cancer Center, Houston, TX, USA

**Keywords:** cancer therapy, CTL, biomarker, CTA, breast cancer

## Abstract

Breast carcinoma is a major health issue for millions of women. Current therapies have serious side effects, and are only partially effective in patients with metastatic tumors. Thus, the need for novel and less toxic therapies is urgent. Moreover, hormonal and antibody therapies effective in other subtypes are not effective in Triple Negative Breast Cancer (TNBC). Immunotherapeutic strategies directed against specific tumor-associated antigens (TAAs) and mediated by specific cytotoxic T lymphocytes (CTL) have been largely underexplored in this disease. Cancer-testis antigens (CTA) are a group of TAAs displaying the ideal characteristics of promising vaccine targets, *i.e.* strong immunogenicity and cancer specificity. The CTA, Sperm Protein 17 (SP17), has been found to be aberrantly expressed in different neoplasms, including ovarian and esophageal cancers, nervous system tumors and multiple myeloma, and has been suggested as a candidate target for immunotherapy.

Here, we evaluated SP17 expression levels in breast cancer cell lines, invasive ductal breast carcinoma, including patients with TNBC, and adjacent non-neoplastic breast tissue, and determined whether SP17 was capable of generating SP17-specific cytotoxic T lymphocytes *in vitro*.

We showed that SP17 is expressed in breast cancer cell lines and primary breast tumors and importantly in TNBC subtype, but not in adjacent non-tumoral breast tissue or unaffected tissues, except in male germinal cells. Furthermore, we detected specific anti-SP17 antibodies in patients’ sera and we generated SP17-specific, HLA class I-restricted, cytotoxic T lymphocytes capable of efficiently killing breast cancer cells.

## INTRODUCTION

The American Cancer Society estimates that approximately 252,710 new cases of female breast cancer (BC) will be diagnosed with and 40,610 women will die from BC in 2017. Available systemic treatment options for BC, including targeted therapies such as those hormone-based or anti-Her-2/Neu antibodies, as well as standard cytotoxic antineoplastic agents, have proven effective in many patients afflicted with this disease [1–4]. Unfortunately, BC is far from being considered a complete curable disease. Metastatic and relapsed BC remain fatal. A sub-type of BC, namely triple negative breast cancer (TNBC) [5] is negative for estrogen and progesterone receptor (ER, PR) and HER-2/neu. Hence, the hormonal and molecular therapies used in other breast cancer subtypes are not effective. These relatively effective interventions are plagued by their related side effects (i.e., myelosuppression, cardiotoxicity, osteopenia) and their inability to cure patients with advanced stage BC and/or intrinsically or acquired resistant disease [6–12]. These factors underline the urgent need for novel therapeutic strategies.

There have been major advances in understanding the underlying molecular basis of BC over the last 20 years, leading to significant improvement in the design of novel therapeutic strategies for this disease. Nevertheless, only a few of those strategies have focused on the discovery of new targets and therapeutic treatments that exploit the activation of cellular immunity against BC. Such immunotherapeutic approaches would take advantage of specific BC-associated antigens capable of eliciting strong and specific cytotoxic T lymphocyte (CTL) antitumor responses. Despite the sound rationale and promise of such a strategy, BC specific immunotherapy has only recently been explored in both animal models and the clinical setting [13–15].

Immunotherapies based on the discovery of BC-specific antigens present significant advantages over traditional BC treatments such as ease of administration, limited toxicity and high specificity against cancer cells. To date, a number of antigens have been tested as potential targets for BC vaccines in animal models and some have been tested in the clinical setting with variable results [13, 16–28]. The main caveats of these interventions have been the suboptimal specificity for BC cells, tissue distribution and weak immunogenicity of the antigens tested.

More recently, a novel and expanding group of tumor-associated antigens (TAAs), known as cancer/testis antigens (CTAs), has raised interest as a potential target for cancer immunotherapy [29–31]. CTA expression was thought to be testis-restricted but several studies have shown significant expression in cancer tissues, as opposed to their non-tumoral counterparts, suggesting a strong association between their expression and the neoplastic phenotype [32–34]. Another important characteristic of CTAs is their marked immunogenicity. This, coupled with their restricted expression in male and female germinal cells and neoplastic tissues, make these molecules ideal candidates for immunotherapeutic targeting. CTA-based immunotherapies have been tested in animal models of neoplasia as well as cancer patients with variable results [35–37]. Interestingly, CTA expression in BC has been found to affect cell proliferation and to correlate with a lack of estrogen receptor expression and poor prognosis making these molecules very attractive therapeutic targets [18, 38–40].

The CTA Sperm Protein 17 (SP17) is a highly-conserved mammalian protein, normally found in germinal cells, where it contributes to sperm maturation, capacitation and acrosome reaction [41–43]. Aberrant SP17 expression has been shown in ovarian and esophageal cancers, nervous system tumors, multiple myeloma and esthesioneuroblastoma [44–48]. Although SP17 mechanisms of action in cancer cells are still poorly understood, it has been shown that SP17 mediates cell-cell adhesion in malignant B-lymphocytes through heparan-sulfate and enhances cell motility and drug resistance in ovarian cancer and esophageal cancer cells [49–51]. Thus, the development of SP17-targeted immunotherapy may not only generate antitumor cellular immunity capable of cancer cell eradication, but also would interfere with fundamental mechanisms involved in cancer cell survival, metastasis and drug resistance mediated by SP17.

To our best knowledge, SP17 expression and potential as an immunotherapeutic target in BC has not been previously investigated. In this study, we evaluated SP17 expression in human breast cancer cell lines, primary female BC, including TNBC, and adjacent unaffected breast tissues. Additionally, we assessed SP17 immunogenicity in terms of eliciting an antibody response in patients and a cytotoxic response *ex vivo*. Our findings will serve as “proof of concept” for the future development of SP17 as a BC biomarker and immunotherapeutic target in this disease.

## RESULTS

**Figure 1:**
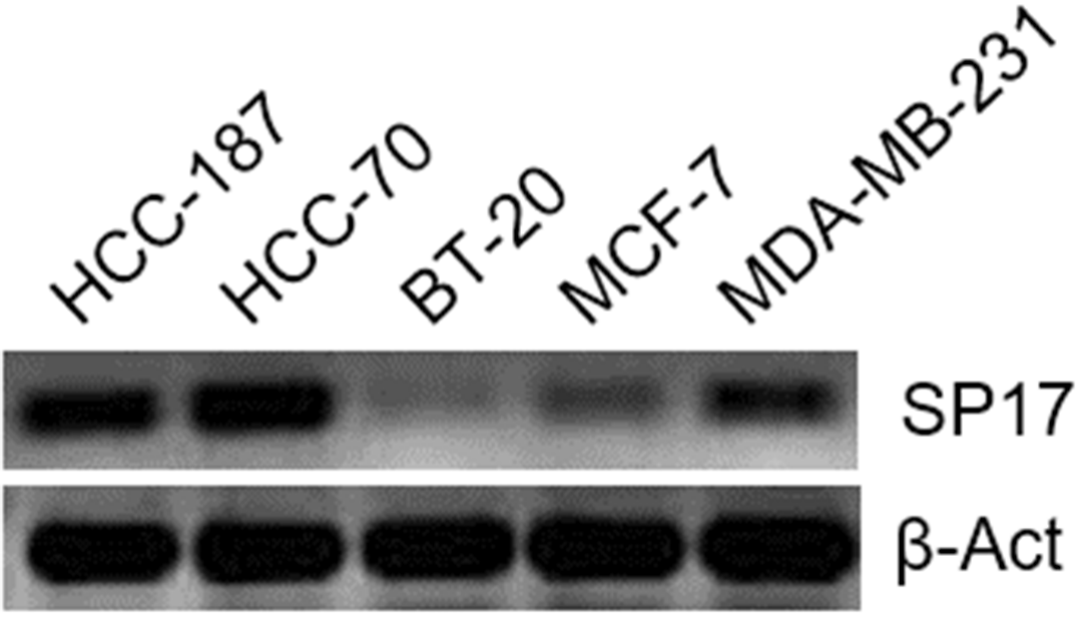
RT-PCR for SP17 in BC cell lines HCC1187, HCC70, BT20, MC-F7, and MDA-MB-231 A reaction without cDNA template (no temp) and with RT reaction performed without retro-transcriptase (no RT) were included as negative controls (not shown). β-ACT expression was used as an internal control.

**Figure 2:**
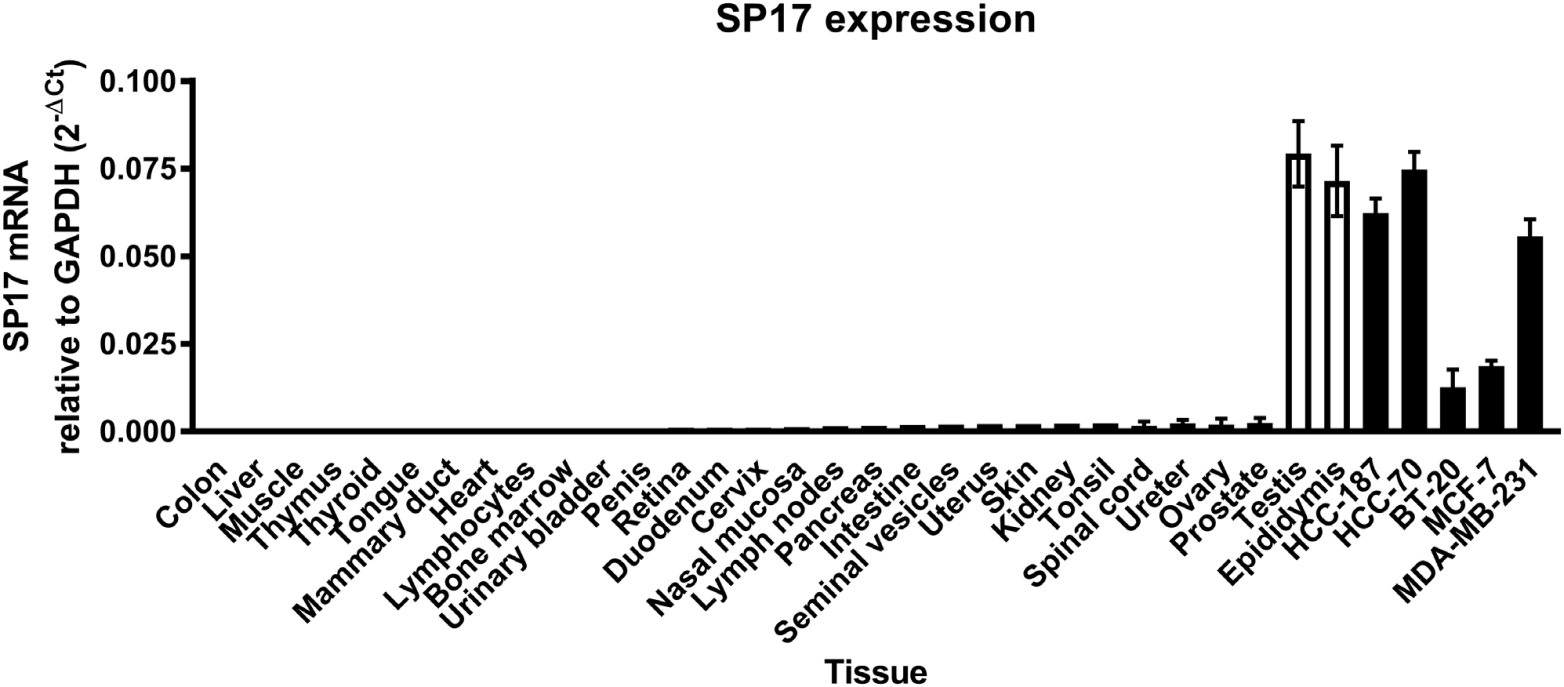
Analysis of SP17 expression in the indicated normal tissues and cancer cell lines Results represents the mean values of the 2^-ΔCt^ calculated out of 3 experiments. Among non-tumoral human tissues, only testis and epididymis showed significant SP17 mRNA expression.

**Figure 3:**
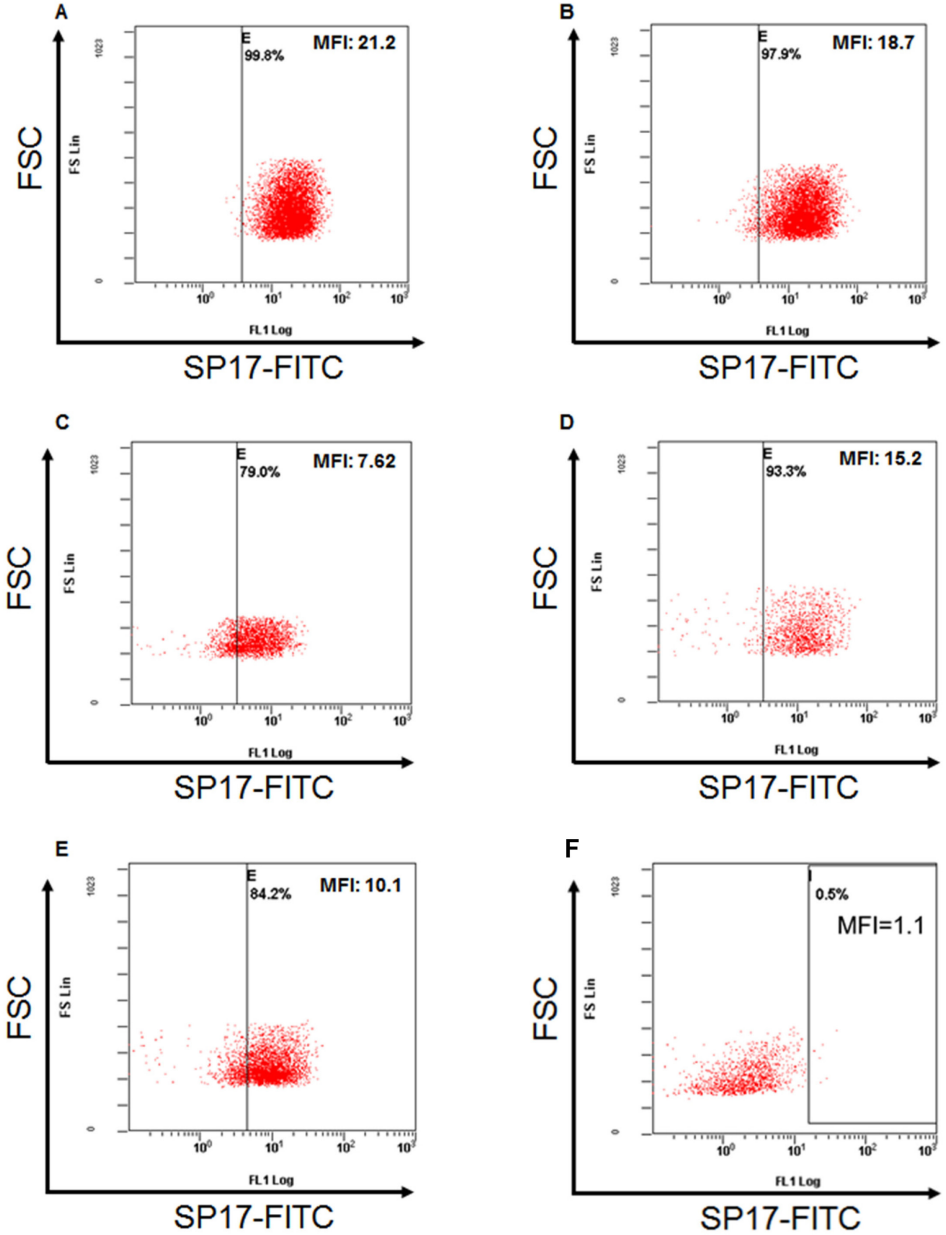
Flow-cytometry analysis for SP17 in **(A)** HCC1187; **(B)** HCC70; **(C)** BT20; **(D)** MC-F7; **(E)** MDA-MB-231; **(F)** non-tumoral human PBMCs (Lonza, Allendale, NJ, USA). After permeabilization as indicated in Materials and Methods, cells were incubated with anti-SP17 mouse monoclonal antibody or with the corresponding isotype control. Results are depicted as the FSC scatter versus FL-1 fluorescence. Vertical bars represent the fluorescent threshold measured on the respective isotype controls.

First, we analyzed SP17 mRNA expression in infiltrating ductal breast cancer (IDBC) cell lines. We detected SP17 mRNA in IDBC cell lines, namely HCC1187, HCC70, BT20, MC-F7, and MDA-MB-231. As shown in Figure 1, HCC1187, HCC70, MC-F7 and MDA-MB-231 displayed higher level of SP17 mRNA expression, compared with BT20. Further, we showed that SP17 mRNA expression was undetectable in a panel of human normal tissues, including the normal mammary duct (Figure 2). We validated our data by analyzing SP17 expression at the protein level in HCC1187, HCC70, BT20, MC-F7, and MDA-MB-231 cell lines by flow cytometry. As shown in Figure 3, SP17 protein was expressed in all IDBC cell lines at different levels. Specifically, HCC1187, HCC70 and MC-F7 showed an expression close to 100% (99%, 97% and 93% respectively), while 79% of BT20 and 84% of MDA-MB-231 cells were SP17+. The MFI values obtained with flow cytometry (Figure 3) correlated with the intensity of the PCR products shown in Figure 1.

**Figure 4:**
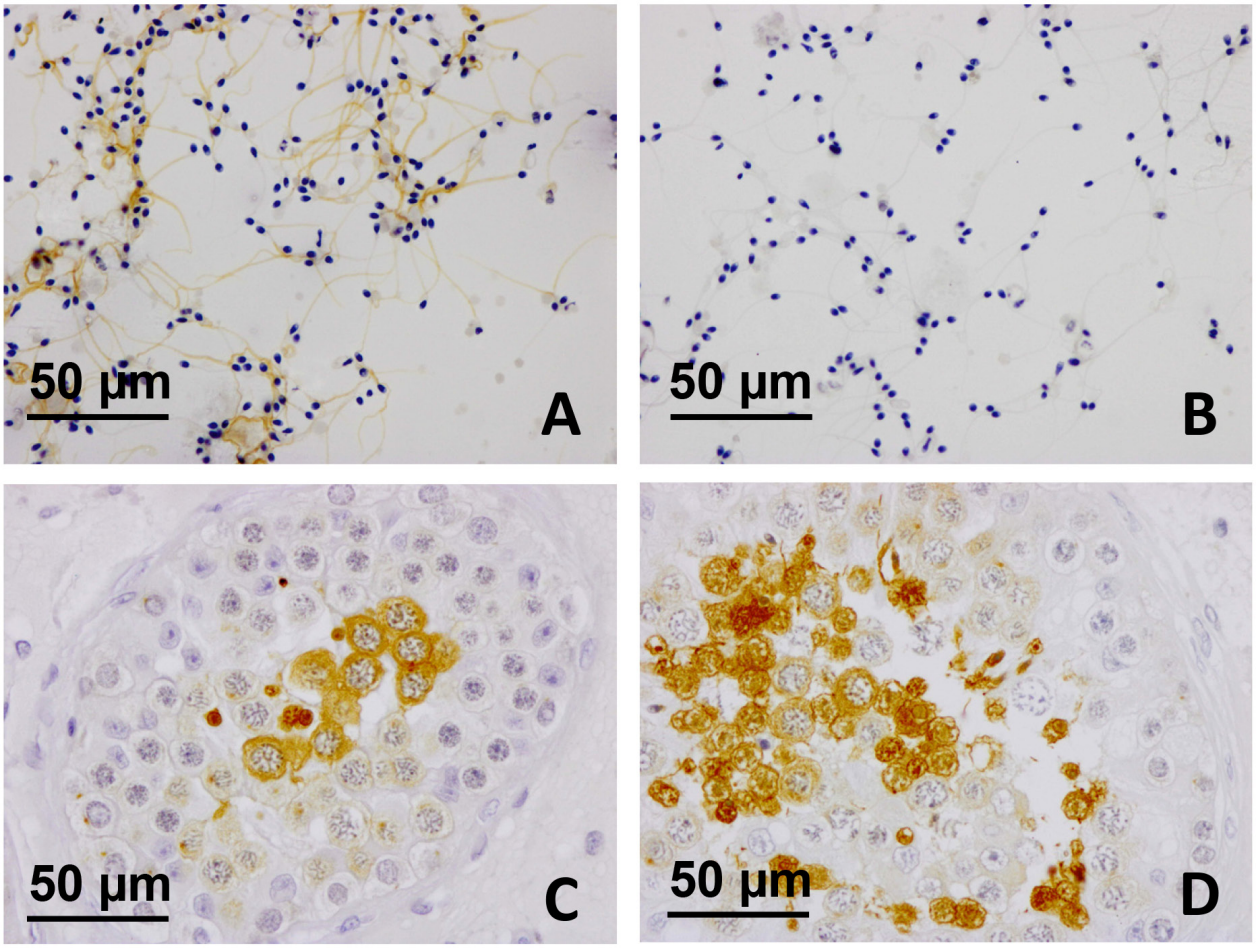
SP17 protein expression in ejaculated spermatozoa and testicular biopsy tissues **(A)** SP17 was found in ejaculated spermatozoa collected from healthy human donors. **(A)** SP17 was localized abundantly throughout the principal piece of the tail (objective magnification 40x). **(B)** The IgG controls were always negative (objective magnification 40x). SP17 was also found in human testicular biopsies **(C-D)**. SP17 was localized in the cytoplasm of some spermatocytes and that of early and late spermatids (objective magnification 40x). Although spermatogonia and Sertoli cells were not labeled, some non-apoptotic spermatocytes and elongated spermatids were immunopositive.

**Figure 5:**
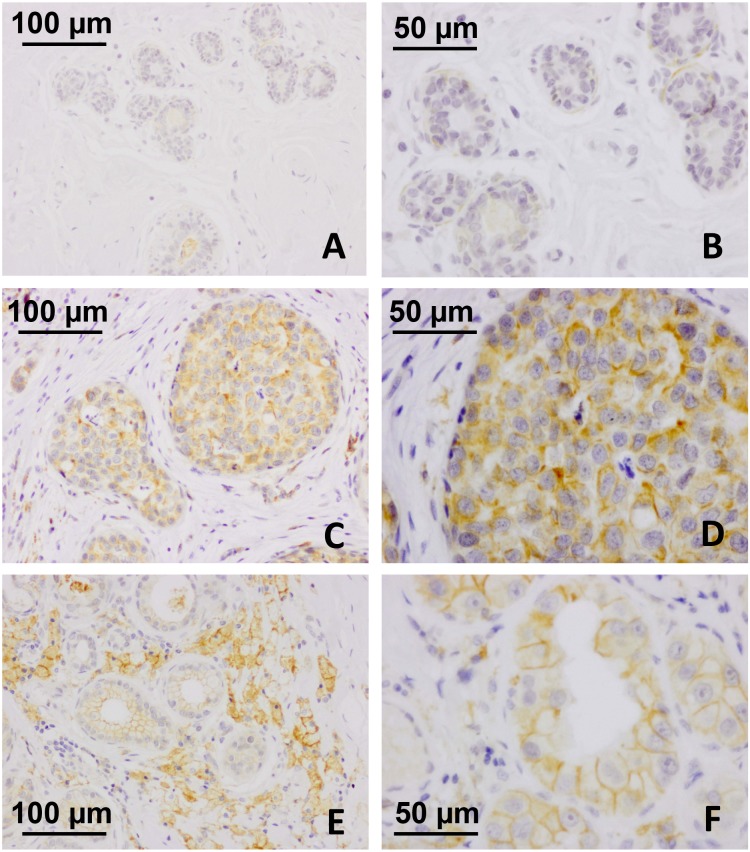
SP17 protein expression in IDBC and TNBC tissues Adjacent non-tumoral breast tissue was found immunonegative for SP17 at low as well as high magnification (**A**, objective magnification 20x; **B**, objective magnification 20x). In contrast, SP17 has been found in infiltrative ductal breast cancers (**C**, objective magnification 20x; **D**, objective magnification 40x), including TNBC (**E**, objective magnification 20x; **F**, objective magnification 40x).

Figure 4A shows the expression of SP17 in spermatozoa collected from healthy human volunteers, and in unaffected testicular biopsies sampled in male subjects for diagnostic purposes (Figure 4C and 4D). Adjacent non-tumoral breast tissue was found immunonegative for SP17 (Figure 5A and 5B). In contrast, SP17 has been found in infiltrative ductal breast cancers (Figure 5C and 5D), including TNBC (Figure 5E and 5F).

**Figure 6:**
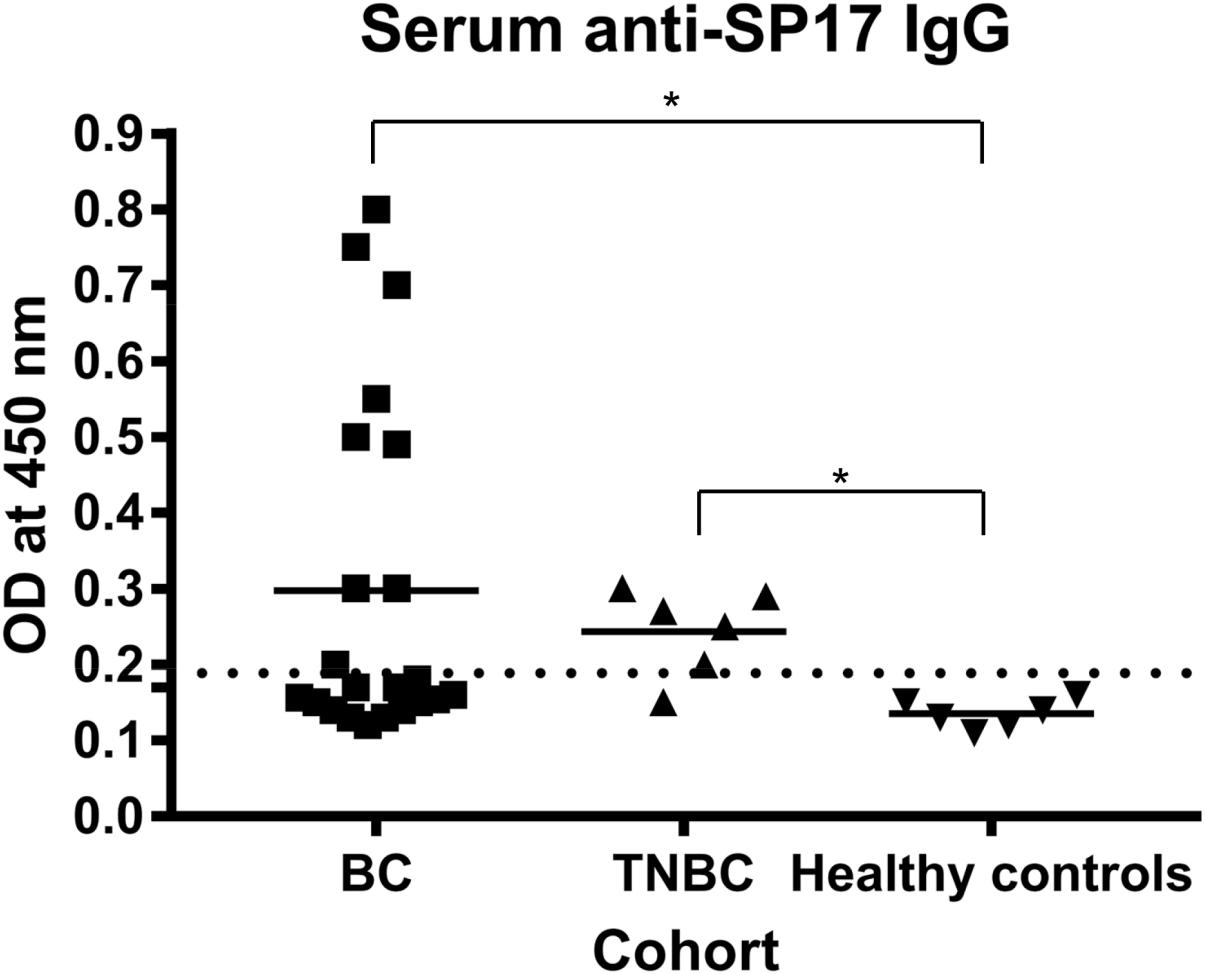
ELISA showed the presence of IgG anti-SP17 in 10/22 BC patients (∼45%) and in 5/6 TNBC patients The right-sided reference interval (OD=0.17, dotted line) was calculated in accordance to the Clinical and Laboratory Standards Institute’s (CLSI) guidelines [64], using MedCalc software. *, one-way ANOVA and Dunn's multiple comparisons test p<0.05.

To evaluate the suitability of SP17 as a target for BC immunotherapy, we determined the presence of anti-SP17 antibodies in the serum of patients and healthy controls by ELISA (Figure 6). One-way ANOVA and Dunn's multiple comparisons test showed that the differences in OD values between HC and tumor groups were significant (p=0.0225 for BC, and p=0.0221 for TNBC). Table 2 summarizes the results from IHC and ELISA.

**Figure 7:**
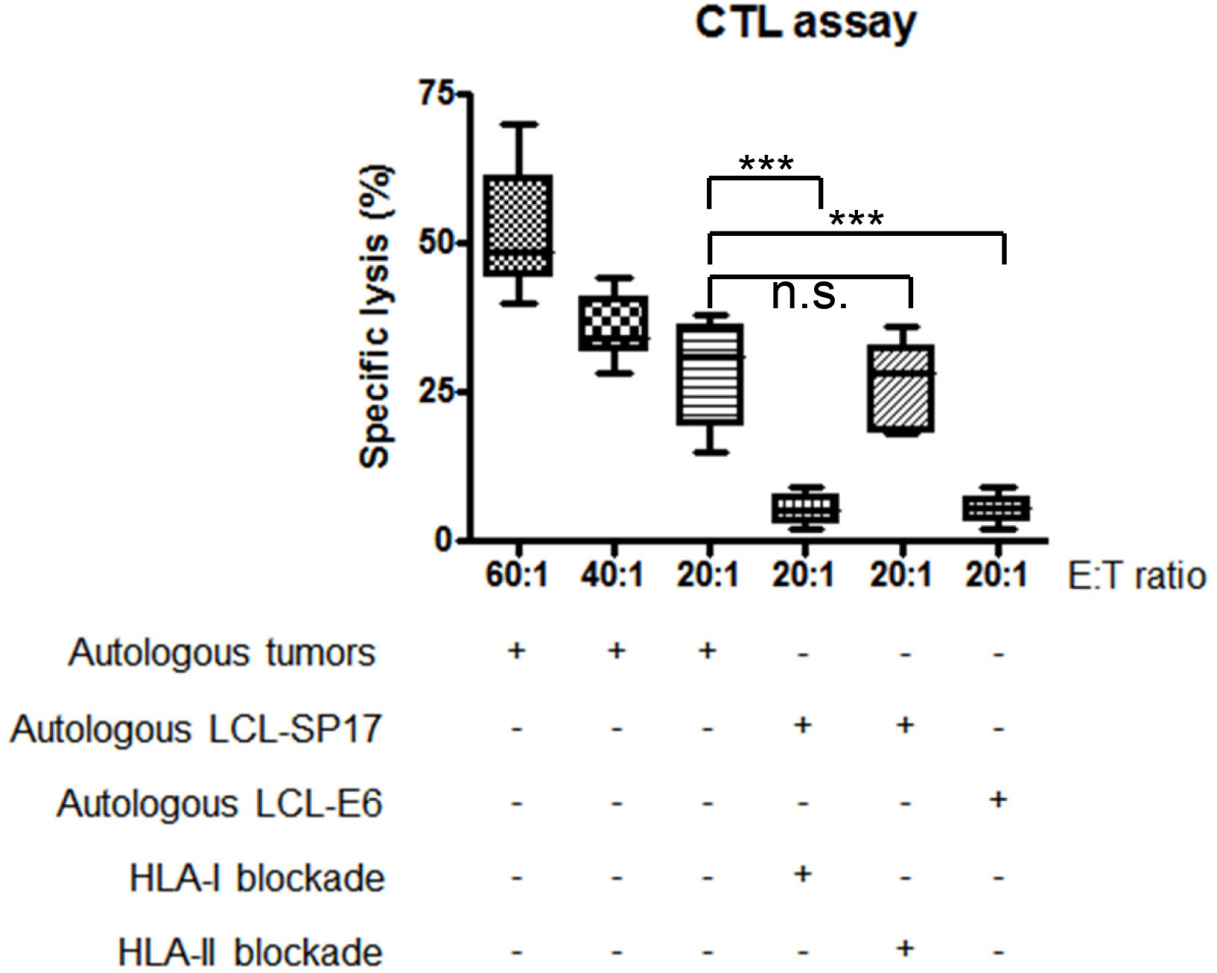
Patients’ CTLs kill autologous tumors in a SP17 and HLA-I restricted manner CTLs generated from 10 BC patients were able to kill autologous BC cells. Experiments were performed in triplicate. Results represent the mean and range values of specific lysis. CTLs were able to kill autologous lymphoblastoid cells (LCL) pulsed with SP17 (LCL-SP17) but not with the unrelated antigen, HPV16-E6 (LCL-E6). Target cell lysis was inhibited by a blocking antibody directed at HLA class I molecules but not HLA class II molecules (HLA-I blockade and HLA-II blockade, respectively). Mann–Whitney U test was performed to assess statistical significance (α=0.05): ***, p≤0.001; n.s.= not significant.

Autologous SP17-stimulated T-cells were able to specifically kill IDBC cells derived from patients’ primary samples (Figure 7). To determine the SP17-specificity of cell lysis, we measured T-cell-mediated killing of autologous LCL, pulsed with HPV-E6 antigen or with SP17. Results (Figure 7) show that significant lysis is detectable only in SP17-expressing target cells, namely LCL-SP17. We also proved that SP17-stimulated T-cells were unable to kill LCL-SP17 targets when HLA-I molecules were blocked through a specific antibody, while killing activity was not affected by HLA-II blocking (Figure 7).

## DISCUSSION

Our findings demonstrated, for the first time, the expression of the CTA SP17 in BC cell lines and primary BC cells, including TNBC. We showed that SP17 is expressed at both mRNA and protein levels in BC cell lines. Importantly, SP17 is expressed in BC cell lines derived from both primary (HCC70, HCC1187, BT20) and metastatic (MDA-MB-231 and MC-F7) BC, indicating that it is conserved during the metastatic process. SP17 expression in metastatic sites is relevant since the 5-year survival rate of patients drops from 98% if diagnosed with localized malignancy to 27% when they are diagnosed with metastatic disease (http://seer.cancer.gov/statfacts/html/breast.html, SEER 18 2007-2013 data). Moreover, we have demonstrated the presence of SP17 protein in BC tissues, including TNBC, but not in adjacent non-tumoral breast tissues, suggesting the specificity of SP17 expression in BC. The specificity of SP17 expression in cancer (aside from its expression in testes) was further supported by the lack of SP17 expression in a wide panel of human healthy tissues, including mammary ducts, as displayed by quantitative RT-PCR.

The current gap that exists in the immunotherapy of BC is the discovery of novel tumor-associated antigens that would allow the development of BC-specific tumor vaccines. Interestingly, SP17 expression has recently been linked to the development of drug resistance in both ovarian carcinoma and esophageal squamous cancer cell lines, indicating this CTA as a potential marker for disease progression and risk of drug resistance in these neoplasms [49, 51]. Moreover, SP17 restricted expression in tumor cells makes it an ideal target for developing new and more effective immunotherapeutic strategies, as previously shown in ovarian cancer and multiple myeloma models [52–54]. The possibility to establish SP17-directed immunotherapies is especially relevant in TNBC, where the widely used antibody, Herceptin [55], is not effective due to the lack of its target, Her-2/Neu [56]. Thus, we evaluated SP17 immunogenicity in BC patients, including TNBC cases.

To this goal, we searched for circulating anti-SP17 antibodies in the serum of BC patients and healthy controls. We detected significant levels of anti-SP17 antibodies specifically in the serum of BC patients, demonstrating its immunogenicity *in vivo*. SP17 antibodies were present also in the TNBC sub-group of patients. Finally, we were able to generate autologous SP17-specific T-cells *in vitro*, which efficiently killed autologous tumors, in a SP17- and HLA-I-specific manner. Due to the tumor-selective expression of SP17, this finding indicates that SP17-based therapeutic vaccines are expected to have minimal toxicities. Immunotherapy is especially and dramatically needed in TNBC. This sub-type lacks estrogen receptor (ER), progesterone receptor (PR) and epidermal growth factor receptor-2 (Her-2/Neu). As such, it cannot be treated with drugs like tamoxifen or trastuzumab, which specifically target hormone receptors or Her-2/Neu. In addition, TNBC is characterized by high recurrence rate and poor prognosis [56]. To date, little progress has been done in the discovery and validation of alternative targets in TNBC. Specifically, MUC1 [57], VEGFR-2 [58], and androgen receptor [59] have been proposed as TNBC targets. Although our study was partially limited to a restricted number of patients and the actual prevalence of SP17 expression in TNBC could not be assessed, SP17 might be a more promising antigen for future therapeutic TNBC vaccine due to its more specific expression in tumor cells, compared to the other aforementioned potential targets.

Our findings support the potential of SP17 as a promising novel target for BC, similarly to what we have previously demonstrated in an animal model of ovarian cancer [52, 60]. These results open the door for the development of innovative, SP17-based immunotherapeutic strategies that may overcome the limitations of available therapies and offer a personalized approach to patients with BC.

## MATERIALS AND METHODS

### Cell lines and primary samples

TNBC cell lines were purchased from the American Type Culture Collection (ATCC, www.atcc.org) and maintained at 37°C with 5% CO_2_ in the complete mediums recommended by ATCC. Table 1 summarizes the characteristics of the cell lines used in this study. HCC70 cells (ATCC No. CRL-2315) were derived from a primary ductal carcinoma; HCC1187 (ATCC No. CRL-2322) were derived from an invasive ductal carcinoma; BT20 (ATCC No. HTB-19), MDA-MB-231 (ATCC No. CRM-HTB-26) and MC-F7 (ATCC No. HTB-22) were derived from metastatic site. Cell lines were analyzed in 2 passages after purchasing, to rule out possible alterations due to *in vitro* propagation.

**Table 1:** BC cell lines characteristics

	ATCC #	Histology	Tumor source	TNBC (yes/no)
**HCC70**	CRL-2315	Primary ductal carcinoma	Primary	Yes
**HCC1187**	CRL-2322	Primary ductal carcinoma	Primary	Yes
**BT20**	HTB-19	Carcinoma	Primary	Yes
**MDA-MB-231**	HTB-26	Adenocarcinoma	Metastasis, pleural effusion	Yes
**MC-F7**	HTB-22	Adenocarcinoma	Metastatic site, pleural effusion	No

A series of consecutive patients with IDBC who underwent surgical treatment and follow-up at the Humanitas Research Hospital, Rozzano, Milan, Italy, were retrospectively collected. For each patient, the histological grade, pathological stage, ER and PR receptors status, HER-2/new, MIB-1 and the number of positive lymph nodes were known. TNBC samples were from the Department of Internal Medicine, Texas Tech University Health Sciences Center (Amarillo, TX, USA). The study was carried out in accordance with the guidelines of the Ethics Committee of the hospital treating the patients, all of whom were fully informed of the possible discomfort and risks of surgical treatment. Table 2 summarizes the characteristics of the subjects included in the study.

**Table 2:** Samples included in this study and summary of IHC and ELISA results

	Number	Median age (range)	SP17 positive by IHC (N of analyzed samples)	Anti-SP17 IgG positive (N of analyzed samples)
**Healthy controls (unaffected adjacent breast tissue)**	7	40 (18-66)	0 (7)	0 (6)
**IDBC**	22	48 (34-62)	10 (22)	9 (22)
**TNBC**	36	50 (38-70)	17 (36)	5 (6)
**All BC combined**	58	48 (34-70)	27 (58)	14 (28)

### RNA isolation and RT-PCR

Total RNA was isolated from TNBC cell lines through the RNAqueous^®^ Total RNA isolation Kit (Ambion, Austin, TX) and 1 μg was reverse-transcribed using the M-MLV Reverse Transcriptase Kit (Sigma-Aldrich, Saint Louis, MO). RNAs from normal human tissues were obtained from FirstChoice^®^ Total RNA (Ambion, Austin, TX). 1/20 of retro-transcription reaction volume was PCR-amplified in 20 μL reaction with PCR Core kit with Taq DNA polymerase (Sigma-Aldrich, Saint Louis, MO). Primers sequences were: SP17 left 5’-GCTCGGAGAGAAAGGAGGTTC-3’, SP17 right 5’-TACTCCCCCATTCTGCTGGA-3’, Human β-actin Real-Time Primer Mix (1 nmol/200 μL, Origene cat. # HP204660).

All reactions were performed at 57°C annealing temperature for 38 cycles with 2 mM MgCl_2_. 10 μL PCR reaction were run in 2% w/v agarose gel stained with ethidium bromide. Pictures were taken after 30 minutes run using the Molecular Imager ChemiDoc XRS+ System (Bio-Rad) equipped with a Quantity One 1-D Analysis Software (Bio-Rad). Quantitative RT-PCR was performed as previously described [33, 61].

### Flow cytometry

400,000 cells were washed with PBS 1X and fixed with buffered paraformaldehyde (4% W/V in PBS, pH=7.4). After 5-min permeabilization on ice with BD cytofix/Cytoperm (BD Biosciences, San Diego, CA), cells were incubated with the anti-SP17 mouse monoclonal antibody that we have previously described [62]. Negative controls were cells incubated with equal amounts of appropriate itsotype control antibody. After 1-h incubation on ice, cells were washed three times in BD Perm/Wash buffer (10X in H_2_O, BD Bioscience, San Diego, CA), and then incubated on ice (30 minutes) with AlexaFluor^®^488 donkey anti-mouse IgG (H+L) (Life technologies, USA). Following 3 washing steps with BD Perm/Wash buffer, cells were re-suspended in PBS and analyzed using a FC500 flow-cytometer (Beckman Coulter).

### Immunocytochemistry

To investigate the immunocytochemical expression of SP17, washed spermatozoa collected from human healthy volunteers donors, were cytocentrifuged for 1 min at 800 rpm on a glass slide using a Cytospin-2 centrifuge and then fixed with Biofix (Bio-Optica; Milan, Italy). The cells were permeabilized with 0.5% Triton X-100 (Sigma), 0.1% sodium citrate in PBS at 4C for 15 min, followed by treatment with either a primary antibody raised against SP17 at RT for 2 hours or with 1 μg/ml mouse IgG1 (DAKO) as a negative control. This was followed by 30-min incubation with the DAKO Envision System. 3, 3’-diaminobenzidine tetrahydrochloride (Dako) was used as a chromogen to yield brown reaction products. The nuclei were lightly counterstained with hematoxylin solution.

### Immunohistochemistry

2 μm thick-sections were cut from formalin-fixed and paraffin-embedded surgical BC or testicular biopsy tissue and processed for immunohistochemistry. In brief, after deparaffining and rehydration, they were immersed in an antigen retrieval bath (Dako, Milan, Italy), incubated with 3% H_2_O_2_ for 15 minutes to quench endogenous peroxidase activity, treated for two hours at room temperature with primary antibodies raised against SP17, or with rabbit IgG (Dako) as a negative control, and then incubated for 30 minutes with the DAKO Envision system (Dako). 3, 3’-diaminobenzidine tetrahydrochloride (Dako) was used as a chromogen to yield brown reaction products. The nuclei were lightly counterstained with hematoxylin solution.

### Enzyme-linked immunosorbent assay

An enzyme-linked immunosorbent assay (ELISA) was performed on the sera of BC patients and of healthy donors. Polystyrene 96-well flat-bottom plates were coated with 5μg/μL SP17 recombinant protein (generated in our laboratory) and incubated overnight at 4 °C. After washing and blocking with SuperBlock^®^ buffer (Pierce, Rockford, IL, USA), plates were placed at 37 °C for 2 hours. Each sample, as well as the negative controls (PBS supplemented with 10% FBS), were diluted 1:1000 in SuperBlock^®^ buffer and incubated for 4 hours at RT. After washing with PBS/0.05% Tween-20, horseradish peroxidase-conjugated goat anti-human IgG (Pierce), diluted 1:5000 in SuperBlock^®^, was added and allowed to incubate at RT for 2 hours. After washing 3 times with PBS/0.05% Tween-20, the 1-Step Ultra TMB-ELISA chromogenic substrate (Pierce) was added to each well for color development for 10 minutes. After blocking the reaction with sulfuric acid, the intensity was measured by the Victor2 micro-plate reader (PerkinElmer, Waltham, MA, USA) at 450 nm wavelength excitation. All samples were run in triplicate.

### Isolation of peripheral blood mononuclear cells and generation of dendritic cells

Peripheral blood mononuclear cells (PBMCs) were prepared by separation of heparinized blood with density gradient centrifugation performed with Ficoll-Hypaque. PBMCs were seeded into 6-well culture plates with 3 mL RPMI 1640 at the density of 8-10X10^6^ cells/well. After 2 hours incubation at 37°C and 5% CO_2_, non-adherent cells were removed; adherent cells were maintained in RPMI 1640 supplemented with 10% FBS, 10^3^ IU/mL interleukin 4 (IL-4) and 800 IU/mL granulocyte-macrophage colony-stimulating factor (GM-CSF). After 1-week culture, dendritic cells (DCs) were harvested and pulsed with SP17 protein [53, 54].

### DC pulsing

DCs were washed twice with PBS and transferred in a 50 mL polypropylene tube. The recombinant SP17 protein (rSP17) was generated in our laboratory [55] and mixed with the cationic lipid DOTAP (Roche, Mannheim, Germany) at room temperature for 20 minutes, then added to the DCs for 3 hours at 37°C.

### Generation of SP17-specific CTLs *in vitro*

Antigen pulsed DCs were co-cultured with fresh autologous PBMCs at a ratio of 1:10 in RPMI 1640 with 2% autologous serum, 10 IU/mL IL-2 and 5 ng/mL IL-7. Irradiated autologous PBMCs feeder cells and SP17 protein (50 μg/mL) were added every 7 days, while IL-2 was added every 3 days [53, 54].

### Cytotoxicity assay

A standard 4-hour LDH release cytotoxicity assay (LDH Release Assay Kit, Promega, USA) was performed to evaluate the cytotoxic activity of the SP17-stimulated T cells. Cytotoxicity against autologous breast cancer primary cells was determined at various effector:target cell ratios. For the measurement of CTL-mediated lysis of autologous lymphoblastoid cells (LCL) pulsed with SP17 or HPV16-E6 antigen, cytotoxicity assay was performed with 20:1 effector:target ratio. LCL were generated from autologous PBMCs as described previously [63]. To determine HLA restricted response, a cytotoxicity assay was performed with or without 25 μg/mL HLA-I or HLA-II (W6/32 or L243 monoclonal antibody, respectively, BioLegend 11080 Roselle Street, San Diego, CA) blocking antibodies (effector:target ratio 20:1).
